# A new experimental method for evaluating the effectiveness of auditory signals under realistic background noise conditions: A randomized controlled pilot study

**DOI:** 10.1371/journal.pone.0344350

**Published:** 2026-04-08

**Authors:** Mako Katagiri, Isuzu Nakamoto, Sayaka Uiji, Tomoko Wakamura

**Affiliations:** 1 Research Division of Product Reliability, Osaka Research Institute of Industrial Science and Technology, Izumi, Osaka, Japan; 2 Department of Human Health Sciences, Graduate School of Medicine, Kyoto University, Kyoto, Japan; Politecnico di Torino, ITALY

## Abstract

This study introduces a new experimental method for analyzing auditory signals in the presence of background noise and identifying sounds that are consistently easy for humans to notice in daily environments. Attention to a signal was inferred from a physiological orienting response, measured as the change in heart rate (HR) before and after the presentation of a test sound in an experimental environment designed to simulate daily life. The test sounds consisted of eight musical sounds each composed of two piano notes at different pitches, and eight complex sounds, each composed of two pure tones. Each sound interval—C + E or C + G#—was recorded at four different octaves, covering the frequency range of 130.8 Hz to 1661.4 Hz. The change in HR was calculated as the difference in the mean RR interval (RRI) over five beats before and after the test sound. The strength of the orienting response (OR) was quantified as the RRI difference normalized by the standard deviation of RRI. An absolute value greater than 2 was considered to indicate the presence of an orienting response. Twenty-two healthy young male participants participated in the experiment during a three-day, two-night stay, which was repeated after a washout period of at least one week. The results showed that OR values were reproducible for 11 of the 16 test sounds. Based on the corresponding OR values, C3 + E3 (musical sound) was identified as a suitable pre-signal due to its calming response (negative OR), whereas C6 + G#6 (complex sound) was identified as a suitable alarm signal due to its tension-inducing response (positive OR). These findings suggest that the OR metric for assessing physiological responses, provides a novel and effective approach for objectively evaluating human reactions to unexpected auditory stimuli, when combined with an experimental protocol that simulates daily life and background noise.

## Introduction

Various auditory stimuli characterize the environment in which we live. The environment in which we live is characterized by a wide variety of auditory stimuli. The term auditory signal refers to any sound intended to convey a specific meaning, such as malfunction warnings, message notifications, or operational feedback [1]. Such signals must always be clearly audible in all situations, are easily distinguishable from other sounds, and are noticeable without causing discomfort when heard repeatedly. To investigate human impressions of auditory signals, participants in previous studies listened to target sounds in a quiet room and described their perceptions using evaluative adjective scales [2,3]. This approach, known as the semantic differential method, has been widely applied. However, impressions of signals formed in such controlled environments may differ considerably from evaluations of the same signals when heard amid the ambient sounds of daily life.

Heart rate (HR) has been widely used in previous studies to assess the responsiveness of fetuses [4] and premature infants [5] to auditory stimuli. In addition, HR has been considered for measuring the effectiveness of music therapy [6] and the impact of noise exposure on humans [7]. Therefore, HR can serve as a surrogate indicator of auditory perception. Hence, instantaneous changes in HR provide a promising means of characterizing auditory signals in real-life environments.

HR varies between day and night and is closely linked to autonomic nervous system activity, suggesting it is regulated by circadian rhythms. Numerous human responses to external stimuli have also been shown to exhibit circadian rhythmicity. Specifically, attention is a key cognitive function essential to human performance [8]. Attention is influenced by homeostatic factors (such as wake time and sleep deprivation) and circadian factors (such as time of day). However, it remains unclear whether circadian rhythms affect human responses to auditory signals.

Therefore, this study integrates the aforementioned insights into a novel experimental approach to characterize auditory signals in terms of their effectiveness and associated physiological responses. To assess effectiveness, test sounds were presented in a controlled laboratory environment that simulated daily life, incorporating realistic activities and background noise. To assess the orienting responses of participants, changes in HR before and after each test sound were examined, with data collected during both daytime and evening sessions to detect diurnal variations. The results showed that the test sounds differed in repeatability and response type (i.e., HR acceleration or deceleration).

## Materials and methods

### Study design

This study was conducted and reported in accordance with the Consolidated Standards of Reporting Trials (CONSORT) checklist (see S1 File).

### Participants

Healthy male participants with no history of hearing disorders were recruited. Before the start of the study, all enrolled participants were confirmed to have normal hearing function using an audiometer (model AA-77A, RION Co., Ltd., Japan). The exclusion criteria included extreme morning or evening types, as determined by the Morningness–Eveningness Questionnaire [9]; mental or physical disabilities, as determined by the Cornell Medical Index [10]; and sleep disorders, as determined by the Pittsburgh Sleep Quality Index [11]. None of the participants were smokers or under medication.

A medium effect size (*d* = 0.5) was used to calculate the sample size, based on Cohen’s conventional criteria [12]. This assumption was made because no prior studies have reported comparable data under similar conditions using auditory stimuli. The required sample size was calculated to be *N* = 34 using G*Power (version 3.1.9.2), assuming a two-tailed test, an alpha level of 0.05, and a statistical power of 0.80.A total of 22 male participants (22.3 ± 1.9 years; range, 19–27 years) were ultimately enrolled. In this study, *N* denotes the total number of randomized participants, and *n* denotes the number of participants included in each analysis. The planned number of participants could not be achieved due to recruitment challenges. While our achieved sample size was smaller than the priori requirement, we intentionally ceased further recruitment because prolonged data collection (spanning different seasons) was expected to introduce extraneous variability and thus dilute the signal of interest. To evaluate this sample size (*N* = 22), we performed intention-to-treat (ITT) and per-protocol set (PPS) analyses as sensitivity analyses. Two participants’ Period-2 entries were missing due to a human error. For the ITT analysis, these missing values were imputed using the median calculated from the available observations of the same participant within Period-2, thereby preserving the distributional characteristics of Period-2 and avoiding information leakage across periods. Median imputation is a form of single imputation and is commonly used as a simple robust approach to handling missing data. In addition, it is less sensitive to outliers and maintains the central tendency of the observed data. [13]. No other imputation was performed. The PPS included only completers with no major protocol deviations and no imputation.

For both ITT and PPS, we applied the same one-way ANOVA model and reported eta-squared (*η²*) as the effect size, with *α* = 0.05. ITT (*N* = 18, with interpolation): *F* (10, 187) = 0.763, *p* = 0.665, *η²* = 0.039 (S2 Appendix). PPS (*n* = 16, completers only): *F* (10, 165) = 0.848, *p* = 0.583, *η²* = 0.049 (S3 Appendix). Effect sizes were small and comparable across ITT and PPS (*η²* = 0.039 vs 0.049), and the statistical conclusions were consistent (both non-significant), indicating that our findings are robust to the choice of analysis population. The observed effect sizes (*η²* = 0.039–0.049) were small to small-to-medium, which are lower than the initially expected *d* = 0.5. However, given the clinical relevance of the outcome, these results were considered reasonable and meaningful.

The experiment was conducted between May and August 2019. Before the study commenced, all participants received a detailed explanation of its purpose and procedures and subsequently provided written informed consent. The study was conducted in accordance with the ethical principles of the Declaration of Helsinki and was approved by the Ethics Committee of the Graduate School of Medicine, Kyoto University (Approval No. C1306-1). Furthermore, the study was registered in the University Hospital Medical Information Network (UMIN) database (registration number: UMIN000028250).

### Randomization and masking

Participants were randomly assigned to two groups. Allocation to trial arms was 1:1, with randomized block sizes of four and six. Each group completed the full experiment twice under different conditions, with a washout period of at least one week between sessions. Because this study involved a sound-based intervention, complete masking of conditions for both participants and researchers was not feasible.

The order and timing of multiple test sounds during the measurement sessions were generated using a random number table. Consequently, the researchers were blinded to the sequence in which the sounds were presented until data collection and analysis were completed.

### Study environment

**Fig 1:**
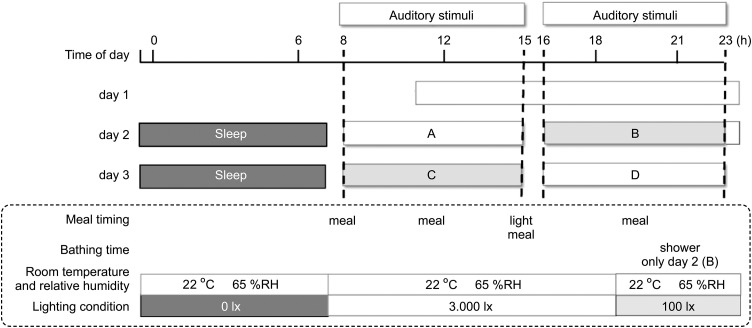
Experimental protocol. Sixteen auditory stimuli were presented in random order and timing within each hour of the experiment, across two of the four designated time segments labeled A, B, C, and D, except for one hour allocated to bathing in segment B. During the experiment, each study group was exposed to the auditory stimuli in either segments A and D or segments B and C. The complete three-day protocol was conducted twice—designated as Period 1 and Period 2—with a washout period of at least one week between them.

Fig 1 shows the experimental protocol. Participants arrived at the laboratory at 11:00 on Day 1 and departed at 23:00 on Day 3. During the experiment, participants went to bed at 23:30 and woke up at 7:30. The daily meal schedule was as follows: breakfast at 8:30, lunch at 12:00, a light meal at 15:30, and dinner at 18:30. Bathing was scheduled for 21:00 on Day 2 only. While staying in the laboratory, all activities—including waking, sleeping, eating, and bathing—were performed according to the established schedule. Unscheduled periods were used for reading or studying. Participants were permitted to bring personal computers into the laboratory and use them until bedtime. The room temperature, relative humidity (RH), and lighting conditions were maintained as follows: 25°C, 65% RH, and 3000 lx from 7:30–19:00; 22°C, 65% RH, and 100 lx from 19:00–23:30; and 22°C, 65% RH, and 0 lx from 23:30–7:30, respectively. Auditory stimuli were randomly presented to both groups during daytime (8:00–15:00) and evening (16:00–23:00) sessions. These four segments, spanning the two full experimental days, were labeled A to D. Participants were prohibited from using personal audio devices while in the laboratory to ensure exposure only to the ambient sounds of daily life, such as eating noises, running water, and typing sounds. To ensure uniform sound characteristics across clothing, participants were instructed to wear the same loungewear throughout the experiment.

**Fig 2:**
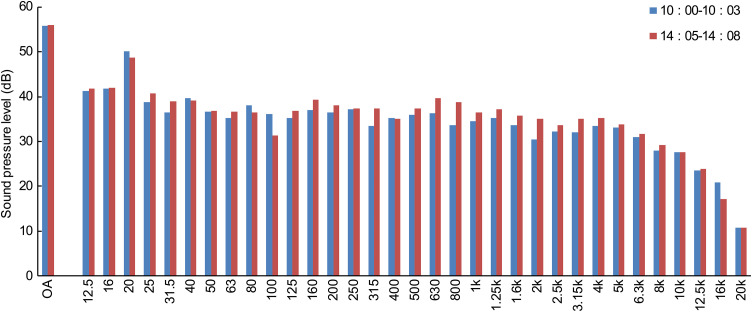
Frequency characteristics of background noise in the laboratory. Third-octave-band distribution of the 3-min Leq. The overall Leq (OA) represents the energy-averaged sound level across all third-octave bands. The horizontal axis indicates the center frequency of each third-octave band, and the vertical axis indicates the sound pressure level in decibels (dB).

To characterize the acoustic environment of the laboratory, background noise levels were measured before the experiment. Measurements were conducted twice a day (morning and afternoon), each lasting 3 min, with the air conditioning operating, at a point near the center of the laboratory. As shown in Fig 2 no notable differences were observed between the morning and afternoon measurements. In addition, the equivalent sound pressure level (Leq) was 56.0 dB in both cases. During the experiment, pink noise matched to the Leq of the stimulus sound was continuously reproduced as background noise by two loudspeakers (VXS3FW, Yamaha Corporation) installed in the laboratory to maintain a constant background noise level.

### Sound stimuli

In this experiment, chords rather than single tones were selected as the test sounds. A chord refers to two or more notes of different frequencies sounded simultaneously. The musical intervals C + E and C + G# were adopted because these chords have been recognized as being easy-to-notice auditory signals [14]. The auditory stimuli comprised two timbral types: “musical sounds,” consisting of two piano notes with short reverberation times, and “complex sounds,” consisting of two pure tones. The audible frequency range of humans is approximately 20 Hz to 20 kHz [15], with the most sensitive range generally between 2 kHz and 5 kHz [16]. Furthermore, the Japanese Industrial Standard specifies that alarm sounds should not exceed 2.5 kHz to ensure that older individuals can hear them clearly [17]. Considering these factors, the test sounds were generated between 130.8 Hz (C3) and 1661.4 Hz (G#6). Specifically, the test sounds were created by increasing the C3 + E3 interval by one octave at a time up to C6 + E6, and likewise for C3 + G#3 up to C6 + G#6. The characteristics of the 16 test sounds (eight musical sounds and eight complex sounds) are summarized in Table 1.

**Table 1:** Characteristics of the eight musical sounds and eight complex sounds.

Musical sound code	Complex sound code	Musical interval frequencies (Hz)	Pitch names
M01	C09	130.8 + 164.8	C3 + E3
M02	C10	130.8 + 207.7	C3 + G#3
M03	C11	261.6 + 329.7	C4 + E4
M04	C12	261.6 + 415.4	C4 + G#4
M05	C13	523.3 + 659.3	C5 + E5
M06	C14	523.3 + 830.7	C5 + G#5
M07	C15	1046.5 + 1318.6	C6 + E6
M08	C16	1046.5 + 1661.4	C6 + G#6

### Procedure

The test sounds consisted of 0.5-s musical or complex tones, each preceded and followed by 0.5 s of silence, and were prepared using Pro Tools (Avid Technology, USA) acoustic processing software. This on/off presentation pattern was based on the method described by Kurakata et al. [18]. The 16 types of test sounds were presented randomly once per hour in each segment (A, B, C, D). therefore, each test sound was presented to the participants a total of 14 times during the experiment. The interval between test sounds was set to 225 ± 30 s, ensuring participants could not predict when the next sound would be played. However, we informed the participants that 16 different short sounds would be presented randomly over the course of one hour.

**Fig 3:**
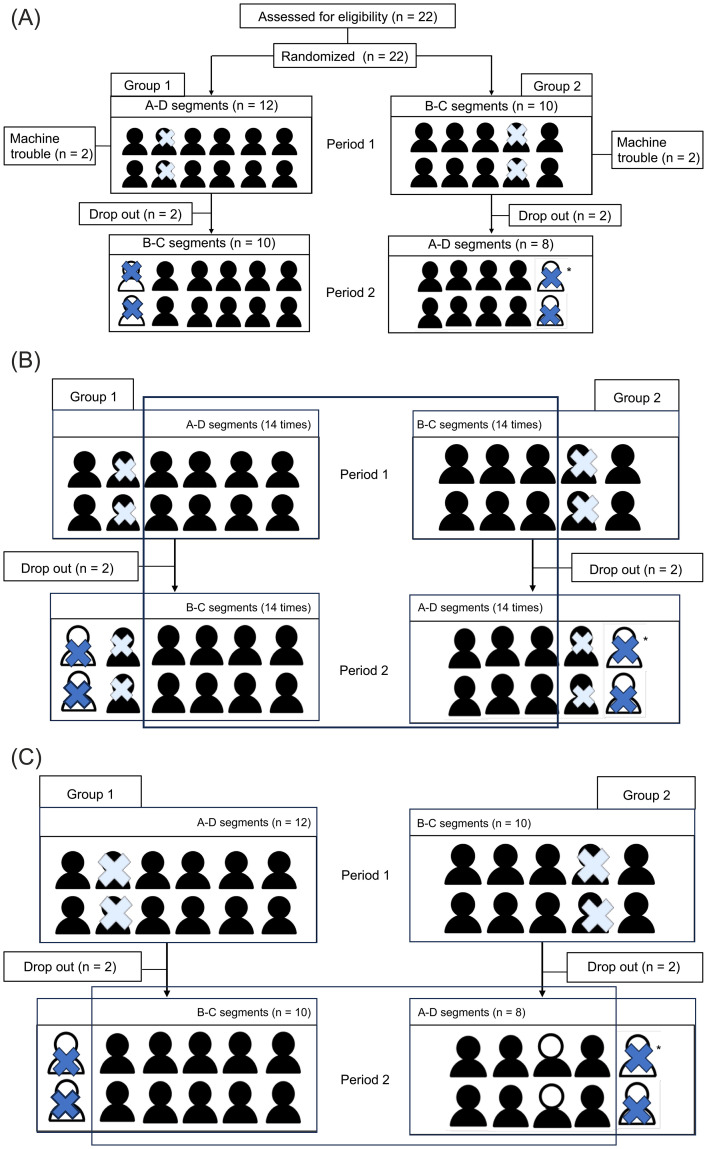
Overview, crossover trial, and randomized controlled trial. **(A).** Participants completed two full experimental sessions in the laboratory, designated as Periods 1 and 2, with a washout period of at least one week between them. During Period 1, data from four participants were unusable due to machine trouble (white crosses). After Period 1, two participants from each group withdrew from the study (indicated by blue crosses). Of the four who dropped out, one participant from Group 2 withdrew consent (*), and the remaining three discontinued participation for personal reasons. **(B).** Participants who completed both Periods 1 and 2 are represented by the overlapping rectangle. The number of participants in Groups 1 and 2 was eight and six, respectively. **(C).** ITT analysis was conducted for the 18 participants, as indicated by the overlapping boxes.

An overview of the experimental design is shown in Fig 3A. The two groups were assigned to receive auditory signals during either segments A and D or segments B and C in Period 1. After a one-week washout period, participants completed Period 2, during which they received auditory signals during the other two segments.

As shown in Fig 3B, a crossover trial was first conducted. The purpose of this design was to determine which test sounds elicited reproducible physiological responses. Test sounds showing significant differences between the two study groups due to a carryover effect (A–D vs B–C within the same period) or a period effect (Period 1 vs Period 2) were excluded from further analysis. Data from four participants in Period 1 were unavailable due to machine trouble. Four participants did not continue into Period 2—one withdrew consent, and three discontinued participants for personal reasons.

As shown in Fig 3C, ITT analysis was conducted using data from the second experimental period, during which participants had become accustomed to the experimental environment. The analysis included 10 participants randomized to Group 1 and 8 to Group 2. After imputing missing values, an ITT analysis was performed.

### Measurement devices

Throughout the experiment, all participants wore electrodes attached using the three-lead method to measure the RR interval (RRI) and carried a portable electrocardiographic (ECG) amplifier (Polyam II A, Nihon Santeku Corporation, Japan). RRI data were recorded continuously from Day 1 until the end of the experiment, excluding sleep periods. To eliminate the influence of bathing on HR, data collected from 21:00–22:00 in segment B were excluded and replaced by interpolation using data from 22:00–23:00.

The ECG data were stored on a personal computer using the Bio-Parameter Real-Time Analysis System (MaP1058, Nihon Santeku Corporation, Japan). All ECG waveforms were visually inspected to confirm the absence of artifacts.

## Analysis

### Physiological evaluation

Animals exhibit an orienting response (OR), observable as a change in HR, when exposed to salient external stimuli [19]. As described in the previous section, HR was used as a surrogate measure of auditory perception. Therefore, to observe the OR in humans, we extracted participants’ RR intervals (RRIs) before and after each randomly and abruptly presented test sound. From these recordings, we calculated the mean (M_1_, ms) and standard deviation (SD, ms) of the five RRIs preceding the test sound and the mean (M_2_, ms) of the five RRIs following the test sound. The OR value was defined as (M_1_ − M_2_) / SD. If the OR value exceeded 2 or was less than −2, the response to the test sound exhibited a marked change and was interpreted as reflecting auditory perception [20]. A positive OR value (> 2) indicated HR acceleration, reflecting physiological states such as tension, defensiveness, or surprise [21]. Conversely, a negative OR value (<−2) indicated HR deceleration, reflecting a relaxed, tension-free response [22]. A dedicated system was developed to extract all stored RRI data around the onset times of each test sound and to calculate OR values automatically (EXPRESSHRV-01; ATR-Promotions, Inc., Japan). For each participant, the proportion of test sounds judged as “easy-to-notice” was calculated as follows: the number of times the OR value was greater than 2 or less than −2 divided by the total number of valid presentations. This proportion was termed the “apparency” of a given sound.

### Statistical methods

The reproducibility of the test sounds with respect to two factors—carryover effect and period effect—was analyzed using Welch’s t-test (two-tailed). Comparisons among the extracted sounds were conducted using one-way analysis of variance (ANOVA), and differences in the timing of auditory stimulus presentation were evaluated using paired t-tests (two-tailed). All analyses were performed using JMP 17.0 (SAS Institute Inc., USA) for predictive analytics. Statistical significance was set at *p* < 0.05.

## Results

### Reproducibility of the responses

We examined the carryover and period effects using data from two time periods (Periods 1 and 2) based on the apparency values of auditory signals that were perceived as noticeable during the 14-hour testing period, which combined the daytime segments (A and C) and evening segments (B and D). The carryover effect was calculated as the mean of the sum of apparency values from Period 1 and Period 2 within each group (Group 1, *n* = 8; Group 2, *n* = 6), whereas the period effect was calculated as half of the difference between the apparency values in the two periods. These two metrics were used to evaluate the reproducibility of apparency measurements (%) for each sound between the two study groups (Group 1 vs. Group 2). The carryover effects of apparency for the 16 test sounds are presented in Table 2 (S4 Appendix). Significant differences between the two study groups were observed for sounds M05, C09, and C11. Similarly, significant differences in period effects between the two groups were found for C10 and C15, as shown in Table 3 (S5 Appendix).

**Table 2:** Carryover effects (comparison between Group 1 and Group 2).

Sound code	Group 1 Mean	Group 2 Mean	Difference in Means	t-value	*df*	95% CI (lower, upper)	p-value
M01	58.04	55.95	−2.09	−0.227	10.22	−22.47, 18.31	0.825
M02	50.89	48.81	−2.08	−0.203	10.13	−24.89, 20.72	0.843
M03	46.43	36.90	−9.53	−0.941	12.00	−31.57, 12.52	0.365
M04	53.57	48.81	−4.76	−0.388	11.63	−31.63, 22.10	0.705
M05	66.96	45.24	−21.72	−2.368	10.06	−42.15, −1.30	**0.039**
M06	63.39	52.38	−11.01	−0.959	11.09	−36.26, 14.24	0.358
M07	63.39	53.57	−9.82	−0.857	11.82	−34.84, 15.20	0.409
M08	50.00	53.57	3.57	0.358	10.51	−18.51, 25.65	0.727
C09	62.50	40.48	−22.02	−2.304	11.23	−43.01, −1.04	**0.041**
C10	51.79	45.24	−6.55	−0.718	11.98	−26.43, 13.33	0.487
C11	61.61	38.10	−23.51	−3.139	10.39	−40.12, −6.91	**0.010**
C12	40.18	36.90	−3.28	−0.496	11.66	−17.70, 11.16	0.629
C13	53.57	47.62	−5.95	−0.639	11.70	−26.31, 14.41	0.535
C14	58.93	55.95	−2.98	−0.235	10.16	−31.08, 25.12	0.819
C15	43.75	65.48	21.73	1.680	9.35	−7.37, 50.82	0.126
C16	58.14	53.57	−4.57	−0.286	11.84	−30.82, 23.68	0.780

Carryover effect: mean of the sum of apparency values from Periods 1 and 2 (Group 1, *n* = 8; Group 2, *n* = 6). Welch’s t-test (two-tailed) was used for all comparisons. Values of *p* < 0.05 are shown in bold.

**Table 3:** Period effects (comparison between Group 1 and Group 2).

Sound code	Group 1 Mean	Group 2 Mean	Difference in Means	t-value	*df*	95% CI (lower, upper)	p-value
M01	0.45	−1.79	−2.24	−0.51	10.20	−11.99, 7.52	0.622
M02	4.91	−2.98	−7.89	−1.57	9.70	−19.11, 3.33	0.148
M03	0.89	−2.98	−3.87	−1.18	11.63	−11.06, 3.32	0.263
M04	2.68	−4.17	−6.85	−1.63	9.08	−16.33, 2.64	0.137
M05	4.02	−2.38	−6.40	−1.51	11.62	−15.66, 2.86	0.157
M06	5.80	3.57	−2.23	−0.76	8.48	− 8.91, 4.45	0.466
M07	4.02	−0.60	−4.62	−0.90	6.72	−16.85, 7.62	0.400
M08	2.68	−4.17	−6.85	−1.25	11.19	−18.89, 5.20	0.237
C09	1.79	−3.57	−5.36	−1.15	9.40	−15.81, 5.09	0.278
C10	10.71	−5.95	−16.66	−3.28	10.36	−27.95, −5.38	**0.008**
C11	−0.45	−3.57	−3.12	−0.79	11.58	−11.81, 5.56	0.447
C12	−0.45	−7.74	−7.29	−1.65	8.84	−17.34, 2.76	0.135
C13	2.68	−2.38	−5.06	−1.11	9.36	−15.34, 5.22	0.296
C14	−1.79	−4.17	−2.38	−0.65	11.89	−10.42, 5.66	0.531
C15	−1.34	−11.31	−9.97	−2.66	11.95	−18.13, −1.81	**0.021**
C16	−0.89	−6.55	−5.66	−1.82	10.24	−12.57, 1.26	0.099

Period effect: half of the difference between apparency values in the two periods (Group 1, *n* = 8; Group 2, *n* = 6). The difference in mean was defined as (Group 2 Mean − Group 1 Mean). Welch’s t-test (two-tailed) was applied. Values of *p* < 0.05 are shown in bold.

From the perspective of reproducibility, seven musical sounds and four complex sounds (M01, M02, M03, M04, M06, M07, M08, C12, C13, C14, and C16) were retained as candidates for easy-to-notice auditory signals. It is noteworthy that reproducible responses were confirmed for seven of the eight musical sounds.

### Randomized controlled trial

The randomized controlled trial was conducted to identify the most noticeable sound among the 11 candidate sounds. We examined whether differences existed in the 14-hour apparency rates of the 11 test sounds during Period 2, when participants were considered acclimated to the experimental environment and thus suitable for evaluation. In the analysis, it was assumed that there were no differences in sound presentation between the A–D and C–B segments, based on the crossover test results (S2 Appendix). Due to human error, data from two participants in Group 2 were imputed, and the ITT analysis was performed as previously noted. No significant differences were observed in the apparency rates of the 11 test sounds across the 18 participants (*F* (10, 187) = 0.763, *p* = 0.665, *η2* = 0.039; one-way ANOVA).

**Fig 4:**
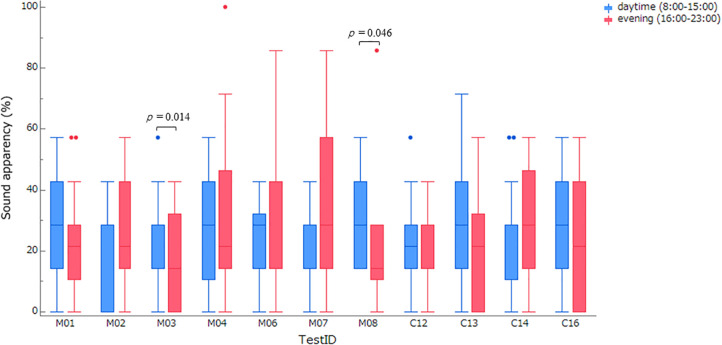
Diurnal variability of apparency. For each of the 11 test sounds, the overall distributions of apparency values observed during the daytime segments (A and C) and evening segments (B and D) are illustrated. The box-and-whisker plots display the median (central line, or upper edge of the box when the median coincides with the 75th percentile), the 25th and 75th percentiles (lower and upper edges of the box), the 1.5 interquartile ranges (whiskers), and individual outliers (dots). The vertical axis represents the percentage of apparency (%). Statistical comparisons between daytime and evening distributions were performed using paired t-tests, with corresponding p-values indicated in the figure.

Next, we examined whether the 11 test sounds that exhibited high reproducibility were equally easy to perceive at different times of day. In the randomized controlled trial, the apparency rates of the 11 test sounds were calculated for all 18 participants, divided into daytime segments (8:00–15:00) and evening segments (16:00–23:00). These results are presented as box-and-whisker plots in Fig 4 (S6 Appendix). Significant differences between daytime and evening distributions among participants were observed only for sounds M03 and M08 (*t* (17) = −2.749, *p* = 0.014, *d* = 0.648, and *t* (17) = −2.152, *p* = 0.046, *d* = 0.507, respectively). These two sounds appear unsuitable for use as auditory signals, suggesting that the time dependence of auditory perception should be considered when designing such signals. In contrast, sounds for which the median apparency rates during the daytime and evening were consistent (both 28%) included M01, M02, M07, C14, and C16. These sounds demonstrated no apparent time dependence. Although further validation of these results is required, this finding highlights the importance of considering temporal consistency when developing easily noticeable auditory signals.

The acceleration and deceleration response rates for each test sound over the 14-hour period were calculated as the number of OR values greater than 2 or less than −2, respectively, divided by the total number of valid sound presentations. As shown in Table 4 (S7 Appendix), sound C16 demonstrated a higher proportion of acceleration (17.8%) than deceleration (11.3%), whereas sound M01 showed a higher proportion of deceleration (16.8%) than acceleration (12.3%). Acceleration and deceleration responses for M02, M07, and C14 were approximately equivalent.

**Table 4:** Ratios of acceleration and deceleration responses for five test sounds.

	M01	M02	M07	C14	C16
Acceleration response (%)	12.3	12.3	14.5	14.8	17.8
Deceleration response (%)	16.8	13.6	13.7	13.6	11.3
Unnoticed response (%)	70.9	74.1	71.8	71.6	70.9

n = 18.

## Discussion

Previous studies have examined the effects of auditory stimuli on physiological functions by analyzing RRIs. These include investigations of the effects of music on stress responses in adult men [23], natural sounds on attention and relaxation [24], environmental sounds on decision-making in individuals with autism [25], mobile phone notification sounds on cognitive function [26], and road traffic noise on HR variability [27]. All of these studies were conducted in laboratory settings designed to evaluate parasympathetic nervous system activity—such as stress and startle responses—under conditions that excluded background noise. The novelty of the present study lies in its evaluation of physiological responses in a controlled experimental environment with realistic background sounds, thereby approximating conditions of daily life.

It has been reported that RRIs can distinguish between different types of responses to stimuli [28]. In the present study, we measured OR values based on RRI changes—specifically acceleration or deceleration—in response to unexpected auditory stimuli and evaluated the apparency of each sound type. To the best of our knowledge, this is the first study to employ chords rather than pure tones as test sounds. Furthermore, we proposed a new method for evaluating auditory signals that can be perceived consistently throughout the day, using the proportion of OR values derived from HR changes in response to clearly noticeable sounds during waking hours.

Table 5 summarizes the profiles of the five test sounds (M01, M02, M07, C14, and C16). Among these, M01, M02, and M07 were musical sounds. Specifically, M01 and M02 had frequencies below 200 Hz—a range in which human hearing is known to be less sensitive [29]. Musical sounds possess rich acoustic characteristics, with energy distributed across a broad frequency spectrum. In contrast, complex sounds composed of monotonous, featureless pure tones were less likely to be noticed. This finding suggests that musical sounds may serve as more effective auditory signals even in environments with high-frequency background noise. In contrast, M07, C14, and C16 exhibited frequencies exceeding 500 Hz, consistent with the bandwidth of auditory sensitivity in adults. Therefore, the results of this study may primarily reflect responses specific to adult participants. M07 was a musical sound that shared the same musical interval as M01 (i.e., C + E), whereas the corresponding complex sound was not selected. This finding implies that the bright and gentle impression associated with the major-third chord [30] may have contributed to the consistent apparency observed for M01 and M07. The musical intervals of C14 and C16 were augmented fifths, a type of interval often described as “muddy” and “unstable” [31], which may be masked by environmental background sounds. However, complex sounds—with their simple, monotonous structures—may have persisted in perception because their reduced tonal ambiguity improved auditory detection at higher frequencies.

**Table 5:** Profiles of the five test sounds.

Test code	M01	M02	M07	C14	C16
**Musical interval frequencies (Hz)**	130.8 + 164.8	130.8 + 207.7	1046.5 + 1318.6	523.3 + 830.7	1046.5 + 1661.4
**Pitch names**	C3 + E3	C3 + G#3	C6 + E6	C5 + G#5	C6 + G#6
**Musical sound code**	О	О	О	×	×
**Complex sound code**	×	×	×	О	О
**Acceleration response**	×	—	—	—	О
**Deceleration response**	О	—	—	—	×

A circle (О) indicates “applicable,” a cross (×) indicates “not applicable,” and solid lines (—) indicate “no difference.”

Fig 4 shows that certain sounds exhibited different characteristics between daytime and evening periods, suggesting that other sounds may follow a similar pattern. Although further investigation is warranted, identifying sounds that demonstrate time-dependent ease of perception, as observed in this study, provides valuable insights for the design of effective auditory signals.

Finally, M01 exhibited a higher rate of deceleration responses compared with acceleration responses, indicating that this sound elicited a physiological response without inducing tension. Conversely, C16 elicited a higher rate of acceleration responses, strongly suggesting an association with tension and surprise. From an applied perspective, M01 could be suitable for use in calm contexts, such as a pre-signal for announcements, whereas C16 could serve as an emergency alert signal to enhance safety and security. These findings highlight the potential for context-appropriate sounds to enhance auditory signaling systems. Furthermore, this approach has the potential to substantially improve the auditory quality of living environments, as it enables the design of signals that account not only for intrinsic perception but also for loudness, timing, spatial context, and situational appropriateness across different settings. Hence, it may contribute to advancing the current paradigm of auditory signal design.

Importantly, the strength of this approach lies in its reliance on physiological measurement rather than subjective evaluation alone. Subjective evaluations are commonly used but can be influenced by individual perception and variability. In contrast, physiological measures, such as heart rate and electroencephalography, provide more objective indicators of participants’ responses. Geangu et al. [32] showed that wearable devices can capture such responses even in infants.

Whether the sounds identified in this study are truly significant—in the view that the characteristics of the sounds are directly related to the nature of the physiological responses they evoke—remains a question that requires further experimental verification. Nevertheless, the experimental method introduced in this study represents a novel and effective approach for investigating the range of sounds that humans are most likely to perceive and for objectively quantifying physiological responses to auditory stimuli. This combined approach to measurement and analysis provides a foundation for developing auditory signals that are less susceptible to masking in daily environments and more likely to elicit desired behavioral responses. Future studies with larger and more diverse samples are needed to validate and extend these preliminary findings.

## Limitations

This study had several limitations. First, only young male participants were included; therefore, the findings may not be generalizable to other populations. Older adults may exhibit age-related reductions in auditory sensitivity, and physiological responses in female participants may vary due to menstrual cycle–related fluctuations. Future studies should include more diverse participant groups to enhance generalizability.

Second, the sample size was smaller than initially planned, which may have limited statistical power. Although sensitivity analyses in both the ITT and PPS populations yielded consistent conclusions, replication with larger samples is necessary. Third, the observed effect sizes were relatively small, possibly due to the specific loudness levels, sound types, and temporal patterns selected for stimulus presentation. Optimizing stimulus parameters may improve sensitivity in future research. Fourth, some test sounds may have been partially masked by background noise, potentially reducing perceptibility. Stricter ambient noise control or sound-attenuated environments may mitigate this issue.

Finally, the auditory stimuli were generated using a piano; different results might be obtained with other instruments or sound sources. Future studies should examine whether the observed effects generalize across different acoustic sources.

## Conclusion

In this study, we evaluated the potential of using OR values derived from RRIs as a novel method for assessing physiological responses to unexpected auditory stimuli. Using this approach, we identified the acoustic characteristics of auditory signals that are easy-to-notice. Specifically, after confirming the reproducibility of the test sounds by examining both carryover and period effects in a controlled laboratory environment designed to simulate daily life, we identified five test sounds that consistently elicited apparent responses and were not affected by diurnal variations. Among these, M01 and C16 demonstrated the greatest potential as effective auditory signals—M01 as a pre-signal and C16 as an alarm signal. These sounds may be considered time-independent, easy-to-notice auditory cues under conditions that approximate real-world environments.

## Supporting information

S1 File Consolidated Standards of Reporting Trials (CONSORT) Checklist. (DOCX)

S2 Appendix Dataset for the intention-to-treat (ITT) analysis of the randomized controlled trial (RCT). (XLSX)

S3 Appendix Dataset for the per-protocol set (PPS) analysis of the randomized controlled trial (RCT). (XLSX)

S4 Appendix Dataset for carryover effect analysis. (XLSX)

S5 Appendix Dataset for period effect analysis. (XLSX)

S6 Appendix Dataset related to Fig 4. (XLSX)

S7 Appendix Dataset related to Table 4. (XLSX)
